# Nanomedicine Ex Machina: Between Model-Informed Development and Artificial Intelligence

**DOI:** 10.3389/fdgth.2022.799341

**Published:** 2022-02-18

**Authors:** Mônica Villa Nova, Tzu Ping Lin, Saeed Shanehsazzadeh, Kinjal Jain, Samuel Cheng Yong Ng, Richard Wacker, Karim Chichakly, Matthias G. Wacker

**Affiliations:** ^1^Department of Pharmacy, State University of Maringá, Maringá, Brazil; ^2^Wacker Research Lab, Department of Pharmacy, Faculty of Science, National University of Singapore, Singapore, Singapore; ^3^Biological Resources Imaging Laboratory, Mark Wainwright Analytical Centre, UNSW Sydney, Sydney, NSW, Australia; ^4^YellowMap AG, Karlsruhe, Germany; ^5^isee systems, Lebanon, NH, United States

**Keywords:** nanomedicine, liposomes, nanoparticles, artificial intelligence - AI, design of experiment - DoE, machine learning - ML, PBPK/PKPD modeling and simulations, drug delivery

## Abstract

Today, a growing number of computational aids and simulations are shaping model-informed drug development. Artificial intelligence, a family of self-learning algorithms, is only the latest emerging trend applied by academic researchers and the pharmaceutical industry. Nanomedicine successfully conquered several niche markets and offers a wide variety of innovative drug delivery strategies. Still, only a small number of patients benefit from these advanced treatments, and the number of data sources is very limited. As a consequence, “big data” approaches are not always feasible and smart combinations of human and artificial intelligence define the research landscape. These methodologies will potentially transform the future of nanomedicine and define new challenges and limitations of machine learning in their development. In our review, we present an overview of modeling and artificial intelligence applications in the development and manufacture of nanomedicines. Also, we elucidate the role of each method as a facilitator of breakthroughs and highlight important limitations.

## Introduction

In ancient theater, the Latin calque “*deus ex machina*”, the “*god from the machine*”, referred to a crane or trapdoor used to suspend objects on stage. Not unlike the unexpected developments in a play, computational aids have the potential to transform the healthcare industry with a strong impact on biomedical research. Model-informed drug development (MIDD) refers to the application of a wide variety of quantitative models derived from preclinical and clinical data to facilitate early decision-making in drug development (1). They were formally recognized by the US-FDA in 2017 as part of their performance goals and procedures commitment letter and involve exposure-based, biological, and statistical models which can give support to establish more successful therapeutic regimens of drug products and increase the chances of approval by regulatory agencies (2).

In drug development, the identification of suitable candidates often begins with a mathematical model establishing relationships between material or product attributes and the *in vivo* response (3). This can be followed by a computer-based simulation of the human population to determine different aspects of the pharmacokinetic and pharmacodynamic responses. Modeling and simulation are often used to support commercial decisions, evaluate risks, and streamline regulatory filings (4). They provide a platform to integrate patient characteristics, easing the experiments by optimizing study parameters, selecting dosage forms, and identifying supportive evidence (1, 5).

**Figure 1:**
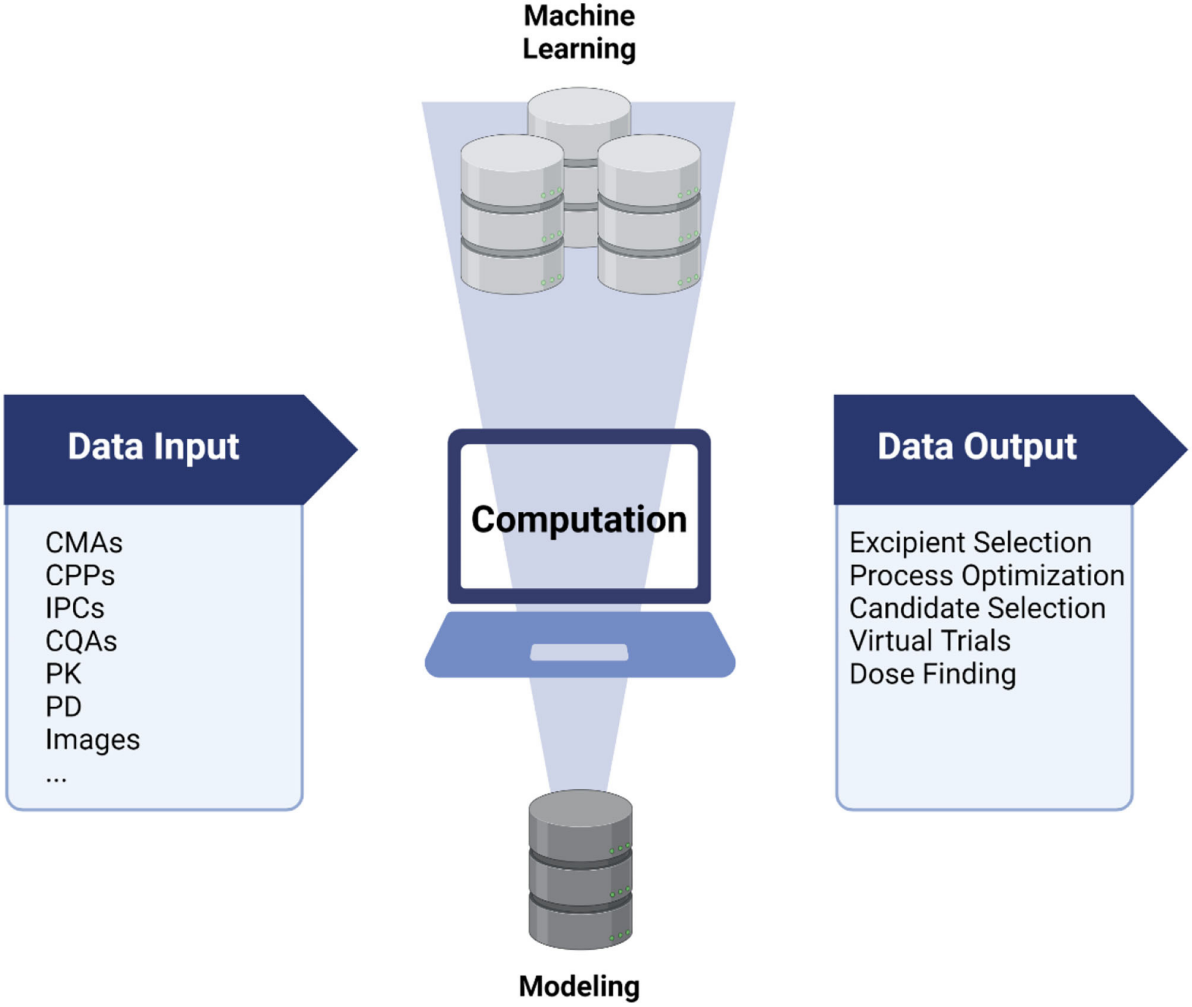
Machine learning and modeling in nanomedicine development include a wide variety of data sources that can be compiled by several algorithms. Machine learning and artificial neuronal networks often require larger data volumes than a conventional modeling approach. Created with www.Biorender.com.

Another evolving area is the formulation and manufacture of drug products. The rise of systems thinking and quality-by-design (QbD) strategies in production led to the evolution of “*computational pharmaceutics*.” Multi-scale modeling techniques and artificial intelligence (AI) together change the face of pharmaceutical sciences by creating virtual process environments and reducing the number of experiments in process optimization (6, 7). By compiling process parameters and quality attributes of drug products in real-time (Figure 1), excipient compositions and manufacturing processes can be optimized (7).

Modeling techniques often contribute to improved process understanding. The integration of critical process parameters (CPPs), In-Process Controls (IPCs), and Critical Quality Attributes (CQAs) enable the quality of the drug product to be controlled. In this context, machine learning (ML) algorithms can play a key role in the identification and prediction of unexpected events and patterns affecting production processes or product performance (Figure 1).

One potential application of computational aids is the development of nanomedicines. They can be broadly defined as products using nanotechnology in the diagnosis, monitoring, and treatment of diseases (8). Among other examples, nanoparticles can be used for diagnostic purposes when they are engineered to target specific tissues and enable anatomical or functional imaging. They may act as contrast agents in computer tomography, magnetic resonance (MR) imaging, positron emission tomography (PET), and other imaging techniques (9). In drug therapy, nanoparticle formulations allow the delivery of one or more drugs (e.g., polymer and lipid-based nanoparticles, micelles) or act themselves as therapeutic agents (e.g., extracellular vesicles, inorganic nanoparticles). Surface-functionalized and stimuli-responsive therapeutics respond to specific stimuli such as a change in pH, enzymes, or exposure to light and may further enhance selectivity (9–11). Recent applications of nanomedicine involve combinations of chemotherapeutic agents encapsulated into the same carrier. They resulted in a considerable improvement of the overall survival and efficacy as compared to free drug control, e.g., in the treatment of acute myeloid leukemia (12–14).

The unique characteristics of nanomedicines including size, morphology, chemical compositions, and surface properties have a strong impact on drug absorption, cell uptake, or the capability to target drug substances to a specific site of action (10, 11). Despite all advantages and the great number of studies on the development of nanomedicines being published every year, only a few nanopharmaceuticals reached the market so far (3, 15, 16). One major reason is their complexity. While most therapeutics mainly affect the absorption rate, nanomedicines may lead to different biodistribution patterns and, hence, to different organ-specific (side) effects. Consequently, the risks associated with a critical failure are a strong incentive for the application of modern technologies, modeling, and simulation in their design and evaluation. With regard to their *in vivo* behavior, while many other formulation approaches can rely on a rich knowledge base, the small market share together with poor accessibility of data requires smart extrapolations and model designs to make their preclinical and clinical outcomes more predictable (3).

In our review, we discuss recent trends and methodologies applied in the production and evaluation of nanomedicines. Further, we will highlight the challenges associated with these modern technologies and outline the opportunities for their future in drug delivery.

## Modeling Techniques and Machine Learning

The statistical and analytical methods applied in nanomedicine have evolved continuously. While pharmacometrics has a long history in the preclinical and clinical sciences, the digitalization of the 1980s and 1990s had a strong impact on drug development. Harmonization of the computational frameworks, as well as the definition of key requirements for drug approval by EMA and US-FDA, is one of the latest developments in this area (17, 18).

Early research on AI dates back to the 1950s, realizing that machines could be “trained to learn” and create new problem-solving strategies instead of following a pre-defined model structure. AI has seen several hypes, often sparked by promising approaches that ultimately failed to live up to the expectations. The most important parallel between the established AI approaches is their limited ability to solve a complex problem without a predefined procedure or program. These problems include perception of abstract similarities, rules, and patterns, reasoning, and decision making. ML is one of many concepts in AI research and describes strategies that generate knowledge from experience (e.g., to cluster or classify elements). In nanomedicine development, the existing formulation or test strategy often provides a predefined theoretical framework for data interpretation while experience is gained in production or during the later stages of *in vitro* and *in vivo* testing.

Recent applications of AI involve artificial neural networks (ANNs) and Deep Learning. ANNs were originally inspired by information processing in nature. They are based on independent neurons that fire when an input signal exceeds a certain threshold. Each neuron has an individual activation function and weighted connections to other neurons. The core approach dates back to the early days of ML but remained a theoretical concept for a long time due to limited data availability, storage capacity, and (parallel) computing power. The rebirth of this concept is directly related to mass data acquisition and the processing on large (virtualized) cloud infrastructures based on multicore processors. Recent developments are supported by the design of hardware supporting ML applications. Selected studies involving AI methods in the development of nanomedicine have been summarized in Table 1.

**Table 1:** Selection of studies involving the application of AI in the development of nanomedicines.

**Area of application**	**Methodology**	**Reference**
Formulation and production	ANN associated with the central composite design and genetic algorithm to predict particle size and loading efficiency	Li et al. (19)
	ANN to identify CQAs and optimize the formulation	Amasya et al. (20)
	ANN to predict particle size and identify the variables with higher impact on this parameter	Youshia et al. (10)
	ML algorithms to predict particle size and PDI and to compare different techniques to prepare nanocrystals	He et al. (21)
Pharmacokinetic and pharmacodynamic analysis	Supervised neural networks and *in vivo* protein corona formation data to predict the half-life, accumulation in organs, and size of nanoparticles	Lazarovits et al. (22)
	AI to establish the optimal drug-dose ratio of a combination of unmodified and nanodiamond derivatives of anticancer drugs	Wang et al. (23)
Image analysis	ML to analyze SEM images of nanofibers and identify manufacturing process defects	Ieracitano et al. (24)
	Genetic algorithm to analyze the morphological characteristics of nanoparticles from electron microscopy images and to identify the presence of impurities	Lee et al. (25)
	ML and histologic slides images of tumors and organs to predict the biodistribution and toxicity of contrast agents- based nanoparticles	Kimm et al. (26)
	Deep learning to accelerate and increase the accuracy in the visualization of gold nanoparticles acting as labeling agents for protein identification and tracking in the cells	Jerez et al. (27)
	ANN to evaluate temporal cellular responses of RNA-liposomes and predict transfection efficiency	Harrison et al. (28)
	ANN to predict internalization of nanoparticles in different cancer cells to classify cancer cell types	Alafeef e al. (29)
	ML, tissue clearing and 3D microscopy to evaluate the distribution of nanoparticles within tumor and micrometastases and predict the nanoparticle delivery according to the pathophysiology of micromestatases	Kingston et al. (30)

In the simplest version, the activation function is binary with only two possible responses, the network itself is unidirectional, and consists of only two layers of nodes (neurons). The input layer and the output layer. The input nodes correspond to the properties of the input data, and the output nodes to those of the output. The size of the ANN is dictated by the problem itself. The “knowledge” in neural networks is mainly captured by the weights of the edges connecting the nodes. During training, ANNs use test data to systematically adjust probabilities for the output of each node. Supervised training uses pairs of input and output data to learn the relationship and probability between those two data clusters. They have been used, for example, to estimate the impact of protein corona formation on carrier half-life (22) or nanomaterial-cell interactions (31). Unsupervised methods commonly use input data for regression or clustering to detect outliers and anomalies. In a hybrid model analyzing the structure of nanofibers, they were used to translate and compress electron micrographs of nanofibers into a code that was further analyzed using ML in a supervised setting (24). Deep Learning describes neural networks that contain hidden layers of neurons. Hidden layers do not influence the operational behavior of the network. Still, the underlying structure of the nodes reveals more information on the way this knowledge was obtained. Deep Learning stores partial patterns in a structured way, to form hierarchies, and to apply them to new tasks. Especially in tasks that require a high degree of transfer, they are superior to primitive ANNs. Deep learning has been used for the analysis of complex spectroscopic data (32). One of the major challenges in AI research is the limited availability of information.

As previously indicated, AI training methods still require "big data” to obtain adequate results. The combination between modeling and AI may solve this problem as prior modeling can help to split the overall problem into several subproblems of lower complexity and thereby reduce the demand for training data. Today, modeling plays a key role in the planning and execution of manufacturing processes as well as in pharmacokinetic (PK) and pharmacodynamic (PD) analysis. When data is a limited resource, theoretical frameworks (computer models) can be used for knowledge generation by using smart extrapolations (3, 33). Although AI is often discussed in a similar context, its role in data sciences is complementary. ML algorithms use a purely “*data-driven”* approach without providing any theoretical framework. The recognition of patterns is unbiased and therefore not necessarily connected to any prior knowledge. In the following, we will discuss the application of model-informed strategies and ML in nanomedicine.

## Applications in Formulation and Production

In the production of nanomedicines, a systematic investigation of the process variables with impact on the quality features of the drug product contributes to improved predictability and reduces the risk of quality-related failure (7, 34, 35). In this context, pharmaceutical development widely follows the principles of QbD. After the definition of the quality target product profile (QTPP) including the dosage form, administration route, and technical features of the drug product, CQAs must be identified. To aid in the selection of excipients, quantitative structure-property-relationship (QSPR) models can be used. They predict structure-based relationships between compounds and excipients. Among others, the Vlife MDS 4.2 builder module can be used (36). These models provide a mechanistic approach in the selection of formulation compositions with desired characteristics and predict mechanisms of drug release by correlating physicochemical properties of polymers (material attributes) with the CQAs of the formulation (37). These parameters are continuously monitored and must remain within pre-defined limits to ensure reliable therapeutic performance. Among other parameters, particle size, encapsulation efficiency, immunogenicity, and zeta potential play an important role (3, 15, 38). As a next step, critical material attributes (CMAs) such as the purity of the drug substances or raw materials used to produce nanomedicines are being defined. Together with selected CPPs such as the number of extrusion cycles in vesicle production, they are the foundation of the control strategy.

To monitor CPP and CMAs, process analytical technologies are used. Various sensors provide the data for process simulation and control (39). A change in the CPPs, CMAs, or CQAs is reported to the process control unit and leads to changes in the production process (40, 41). A recent review article discusses some of the most common CQAs, CPPs, and CMAs in the development of nanopharmaceuticals (41).

**Figure 2:**
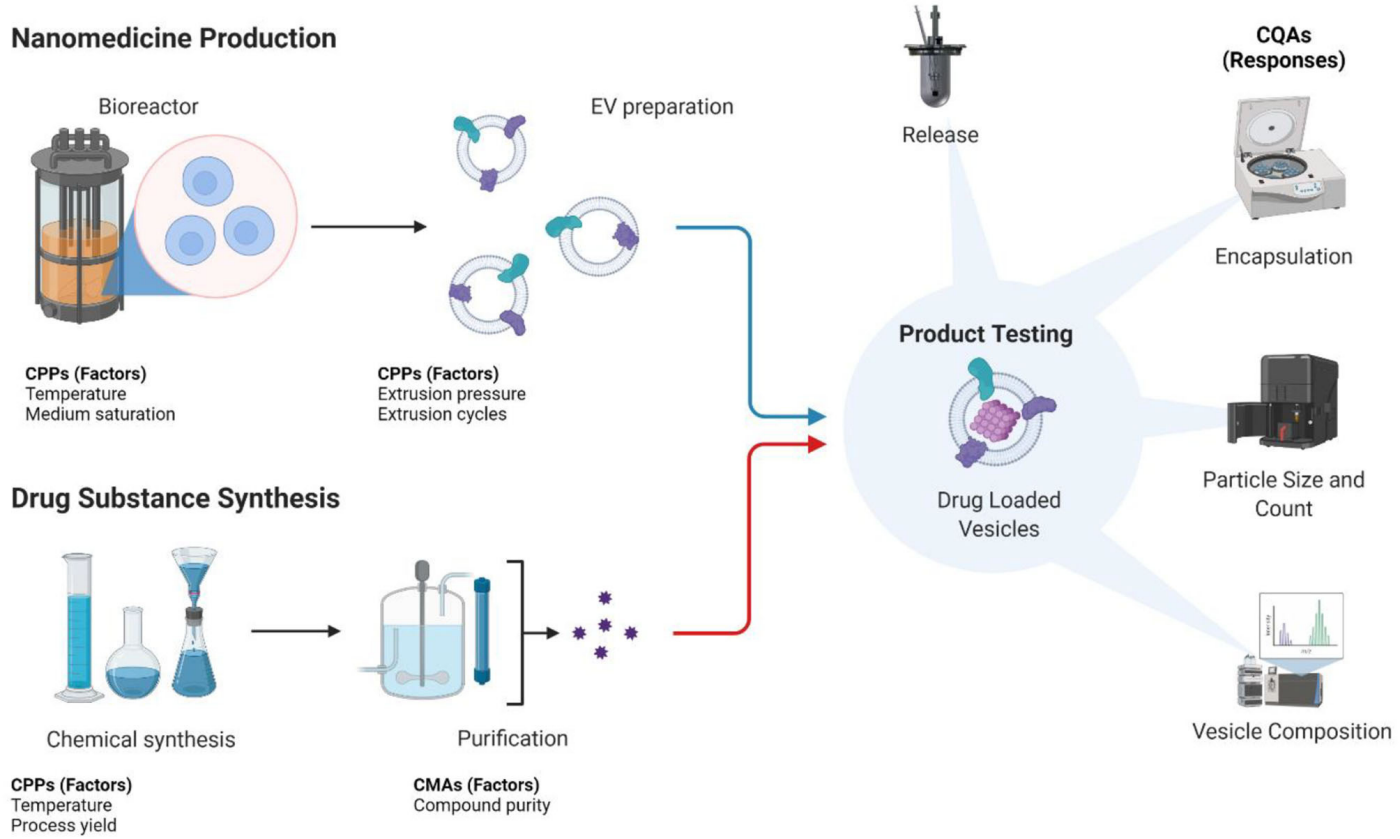
Illustration of nanomedicine production of a drug-loaded extracellular vesicle preparation using a design of experiments approach. Potential CPPs, CMAs, and CQAs are highlighted. Created with www.Biorender.com.

During process validation Design of Experiments (DoE) represents an important tool of QbD strategies. It involves mathematical models correlating the process variables (CPPs and CMAs) with the measured responses (CQAs). This facilitates predictions and process adjustments during production. The control strategy ensures that the product matches the characteristics described by the QTPP (42). The designs applied in DoE studies can be divided into screening designs, such as Plackett-Burman and fractional factorial designs. These are used to compile many process variables (CPPs and CMAs) with an impact on the product characteristics in parallel. For comparison, the response surface methodology includes the central composite and Box-Behnken design. These two examples operate with a smaller number of process variables but explore each parameter at a higher resolution (43), for example, by including the testing of multiple incubation temperatures or carrying out multiple extrusion cycles. An illustration of the manufacturing process of biohybrid vesicles, together with potential CPPs, CMAs, and CQAs is presented in Figure 2.

Three-dimensional plots illustrate the responses obtained from the mathematical models and visualize the relationships between variables and responses. Many studies can be found in the literature using DoE techniques for optimization of nanoformulations (44–50). The accuracy of these predictions as well as the safety levels achieved with superior process controls have attracted the attention of Big Tech companies as well as regulatory agencies (7).

In recent years, ML has been used in product design as well (35, 51). It is a branch of AI that focuses on algorithms and statistical models that recognize, analyze and draw inferences from complex data patterns. The algorithm evolves through undirected evolution and is solely driven by the data. The ANNs, large clusters of computing units, imitate human learning and gradually improve accuracy by repeated analysis of data. They are mainly applied for the prediction of nanoparticle properties (35). When compared to the response surface methodologies using a pre-defined statistical model, better predictions were obtained by ANNs (11). Li et al. (19) combined an ANN with a central composite design and a continuous genetic algorithm to predict particle size and loading efficiency of polymer-lipid nanoparticles containing verapamil hydrochloride. The ANN considerably improved the data fit as compared to the central composite design (19). Another study investigated solid lipid nanoparticles and nanostructured lipid carriers loaded with 5-fluorouracil. After an initial set of experiments, the data was used to train different ANNs and to identify specific CQAs for optimized transdermal delivery (20). The ANN model was successfully used to predict optimal formulation properties (20). Youshia et al. used an ANN to predict the particle size of nanoparticles prepared with commonly used polymers, such as polylactide-co-glycolide (PLGA), polycaprolactone (PCL), ethyl cellulose (EC), and polylactic acid (PLA). The model was able to predict the particle size with a percentage bias of 2, 4, and 6%, for the training, validation, and testing data, respectively. Moreover, it identified input parameters with the strongest impact on the particle size (10).

Going beyond these individual case studies, a systematic investigation was carried out for nanocrystal manufacture including top-down and bottom-up production (21). A total of 910 data sets reporting the particle size and 341 data sets reporting PDI were obtained from different studies. They included nanoparticle preparation methods such as wet bead milling, high-pressure homogenization, and solvent deposition. These data sets were analyzed using eight different ML algorithms. Among them, the light gradient boosting machine (LightGBM) presented a high predictive power with regards to size and PDI for milling and high-pressure homogenization methods. Factors with higher influence on these methods were the milling time and cycle index (21).

On the one hand, these computational models have great potential and will decrease the time and costs of drug development (52). On the other hand, it is not very surprising that highly flexible and adaptive algorithms often achieve improved data fits for well-studied and well-understood process parameters and relationships. Also, the observations widely rely on the quality of the data obtained from the production process. Therefore, ML algorithms are not a replacement for efficient process monitoring. The pressure sensors and responses obtained from the manufacturing site are the eyes and ears of the ANN.

## Applications in Pharmacometrics

The term “pharmacometrics” first appeared in the 1970s (53) and describes a branch of biomedical research concerned with mathematical models of biology, pharmacology, disease, and physiology used to describe and quantify interactions between xenobiotics and patients (54). In this context, the model structure reflects mechanistic relationships between the drug and the living system. Multiple data sources are compiled by human or artificial intelligence.

## Pharmacokinetic Analysis

**Figure 3:**
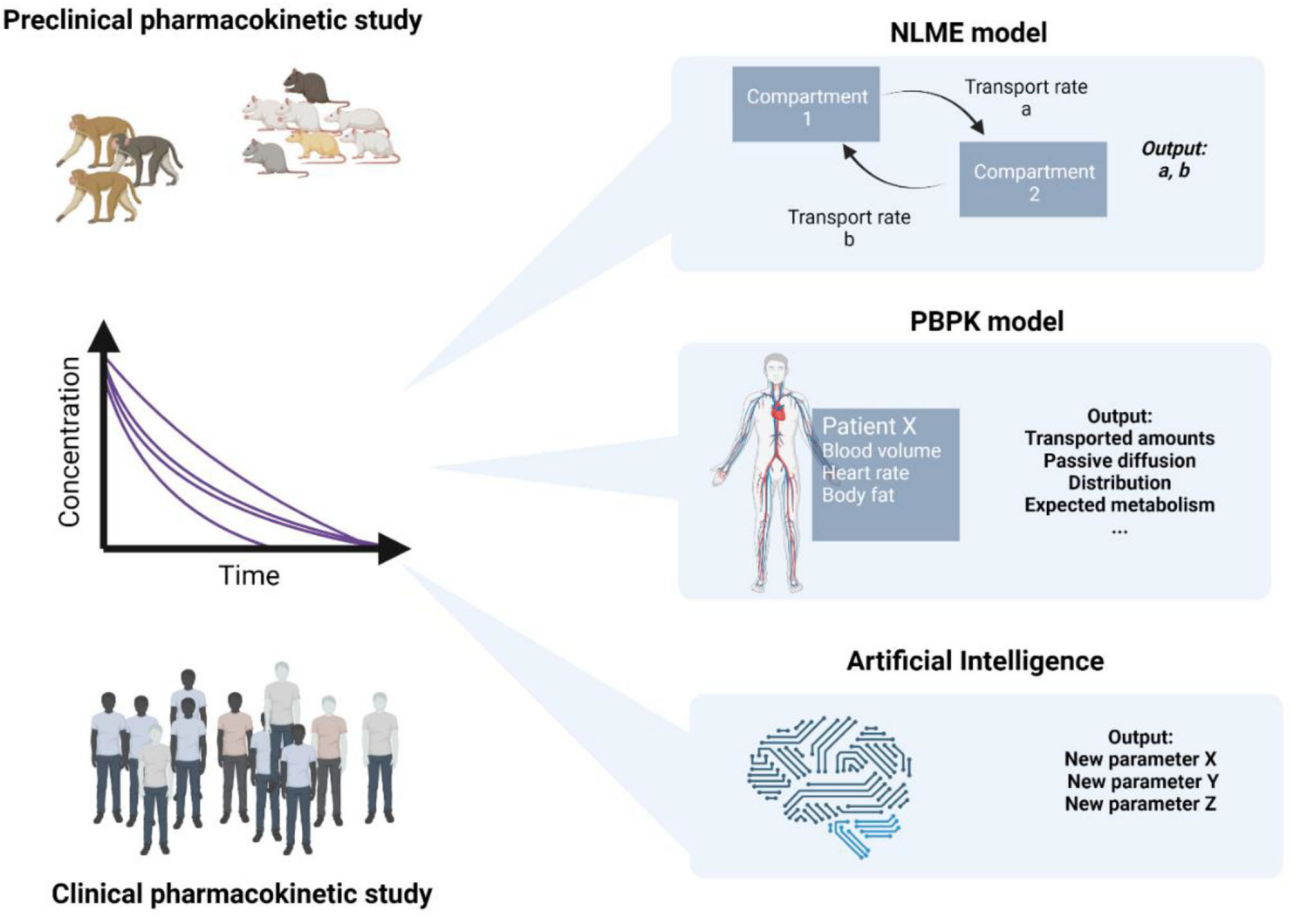
Illustration of the evaluation of pharmacokinetic data using NLME and PBPK models as well as ANNs. Created with www.Biorender.com.

Although a rising number of nanomedicines have been tested in phase-I clinical trials, the number of patients and drug products is relatively limited as compared to other research areas (3). Today, modeling techniques play an important role in the description of physiological processes involved in drug absorption, distribution, metabolism, and elimination (ADME). While traditional PK analysis using population modeling techniques provides a mathematical framework primarily describing the clinical data (Figure 3), physiologically based pharmacokinetic modeling (PBPK), and physiologically based biopharmaceutics modeling (PBBM) include knowledge on physiological processes such as blood flow, lymphatic drainage, or transport mechanisms.

On the one hand, many PK studies have been carried out in nanomedicine and were analyzed by conventional means (55–59). A common method is non-linear mixed effects (NLME) modeling describing non-linear relationships using a limited number of population parameters and parameter variability. On the other hand, there are many PK models and software solutions offered for the analysis of drug products. These computational aids were used to define relationships between CMAs and CQAs of nanomedicines and the plasma concentration-time profile. In this context, the concentration of the drug in the blood plasma serves as a surrogate for bioavailability (3). An illustration of the different model structures found in PK modeling is presented in Figure 3.

Various software packages are offered for modeling and simulation. Among others, they include Monolix® Suite (www.lixoft.com) for NLME models. Monolix® provides a widely non-physiological modeling framework describing the measured drug concentrations of drugs based on random effects including the interindividual differences and fixed effects such as the population-related average and distribution parameters. The operator can include physiological parameters but there is no predefined database of well-characterized physiological processes provided. For example, Monolix has been used to model the PK of the clinical formulation candidates NanoCore-6.4 and NanoCore-7.4 based on a preclinical PK study in Wistar rats. The hybrid model combined the physiological plasma volume with a conventional NMLE model to describe the PK of these PLGA-based carriers (60). Monolix requires the operator to define the transport processes and model structure using the programming language Mlxtran. Alternatively, the visual programming language Systems Thinking, Experimental Learning Laboratory with Animation (STELLA®) can be used. STELLA offers a structured user interface for the description of transport processes. The software can be used to simulate manufacturing environments as well as pharmacokinetic processes. First applications using STELLA for the simulation of nanoparticle delivery include the physiologically based nanocarrier biopharmaceutics model (33, 61). The model identifies an important CQA, the drug release rate from clinical data and establishes a relationship between the *in vitro* and the *in vivo* data. An accurate model fit was confirmed including a wide variety of nanomedicines. Later the model was used to establish *in vitro-in vivo* correlations for liposomal nanocarrier formulations (61). Earlier model designs were applied to nanocrystal formulations and liposomes as well (62, 63). A PBPK-based alternative is offered by GastroPlus™ (www.simulations-plus.com), PK-SIM® (www.systemsbiology.com), and Simcyp^®^ (www.simcyp.com). GastroPlus™ was applied to simulate PK profiles of nanoformulations *in silico* (64). PK-Sim® has been developed by Bayer Technology Services GmbH before it was made open-source. It is designed for users with minimum mathematical and modeling experience through the use of several in-built whole-body PBPK model structures (65). The software was applied in several PBPK modeling approaches to describe the biodistribution and elimination of liposomes (66, 67). So far, combinations with ML or AI have not been reported. Simcyp^®^ traces its roots from a drug-drug interaction tool and has evolved into a sophisticated population-based *in silico* ADME simulator (68). Simcyp^®^ provides users with a comprehensive database of system parameters and pre-coded PBPK model structures (69). There are selected physiologically-based models for nanomedicines available (70).

While the majority of clinical studies are analyzed using conventional PK models, PBPK approaches are published more rarely. The examples reported for liposomes often rely on animal data due to the limited accessibility of biodistribution studies in humans (71–73). Whole-body distribution models are often validated by comparing the observed and the simulated blood plasma concentration only. This leads to a high uncertainty with regards to the accumulation, elimination and, release of nanomedicines and makes accurate predictions more challenging. Hybrid models integrate physiological with non-physiological compartments and often provide useful information without overestimating *in vitro* effects. They are particularly useful when exploring the interplay between CQAs and PK of nanomedicines (3). Also, the application of ML and ANNs is limited by the quantity of the PK data. Consequently, smart model designs and extrapolations dominate current research and can be applied more successfully.

## Pharmacodynamic Analysis

While PK studies provide continuous time-resolved data of drug concentrations in various compartments of a living organism, PD studies comprise *in vitro* and *in vivo* data. On the one hand, pharmacological assays include an investigation of the effect of drugs on target tissues, cells, or biochemical cascades. These assays sometimes come with a high level of sophistication but may also qualify for robust approaches such as high-throughput screening. Many of these methods have been established in the drug discovery pipeline for many years. Their application to nanomedicines is another application of compound screening techniques using drug delivery system libraries instead of drug libraries (74). However, they create a significant volume of data, offering new opportunities for the training of ANNs (74).

Vyxeos^®^ is a dual-drug liposomal product comprising daunorubicin and cytarabine at a fixed molar ratio. This molar ratio was found *in vitro* using a screening approach (75, 76). Nanomedicines play a key role in achieving this exact molar ratio at the target site by providing a suitable tool for co-delivery of multiple compounds in fixed combinations using one drug delivery system (75, 76). Another important area with an impact on nanomedicine is their toxicological characterization. Quantitative structure-activity relationship (QSAR) models are regression models capable of predicting biological or toxicological properties of drugs and drug delivery systems based on their chemical structure. They have been tested for nanoparticles as well (77). *In vitro* and *in vivo* toxicity studies together are essential to comprehend the mechanisms involved in their toxicity. However, chemical characterization and *in vitro* screening tools can be used for the characterization of pharmacological and toxicological responses. Again, the data quantity generated by these methods enables the application of ML algorithms (78).

In the *in vivo* setting, PK and PD data are gathered together, thus quantitative PK/PD relationships may be established and enable assessing the clinical efficacy and/or safety of drug products (79, 80). The clinical efficacy of nanomedicines depends on specific physicochemical characteristics that alter the PK parameters of the drug as well as the interactions with the biological system (77, 81). This highlights the importance of combined *in vitro* and *in vivo* evaluation. While the *in vivo* system is characterized by a wide variety of overlapping processes and allows an estimate of the variability and robustness of the pharmacological effects, *in vitro* studies often provide mechanistic insights at high resolution. Some authors believe that a better understanding of the molecular mechanisms in the body will facilitate the development of more sophisticated modeling and simulation tools for nanomedicines (67). However, this evolution must include both, *in vitro* and *in vivo* methods. At present, the vast majority of studies focus on the development of more complex multidimensional *in vitro* models and tissue engineering without establishing *in vitro-in vivo* relationships. These approaches often fail to represent the clinical realities. In fact, many simple PK/PD models (e.g., standard Emax models) encounter difficulties in estimating the complexity of the *in vivo* responses observed with nanomedicines (82).

The evolution of nanomedicines began with the development of anticancer treatments. As a consequence, patient stratification plays an important role in the design of efficacy studies. Today, improved connections between physiology and disease progressing, along with the effects of the drug on both may be achieved with the use of “*omics”* data. These approaches integrate the identification and quantification of molecules in biological systems on multiple levels. They include genomics, transcriptomics, proteomics, lipidomics, metabolomics, glycomics, metagenomics, microbiomics (83). Important for the development of computational aids is the quantity of data generated. On the one hand, “*omics”* enables the integration of more information on the physiological or pathophysiological state of individuals and patient populations (84). On the other hand, “*big data*” qualify for the application of ML algorithms and ANNs (85). Huang et al. discuss the application of ML using genomic data sets. Synergistic responses to drug combinations in a specific subset of patients may enable a better selection of patients in the clinical trials and, hence, provide patients with more personalized treatments (86).

In summary, PD characterization of nanomedicines enables the integration of modeling and ML tools to a much larger extent. While PK profiles are the result of multiple overlapping processes and require a living organism for data collection, *in vitro* profiling tools generate “*big data”* on the interactions of nanocarriers with cellular or non-cellular assays. Hence, they can be analyzed using ML or ANNs. However, the limitations of the generated data sets are reflected by data analysis as well. Without integration of considerable *in vivo* data, *in vitro* screening often overestimates or underestimates relevant pharmacological or toxicological effects.

## Image Analysis

Another interesting application of ML and ANNs in drug development is the computation of complex images. Imaging methods are widely applied in early research to study material properties of nanomedicines or investigate their biodistribution in cells, tissues, animals, or humans. For example, a genetic algorithm was able to analyze the morphological characteristics (shape, structure, and size) of more than 150,000 nanoparticles from TEM images with high accuracy (>99%) and, in addition, to separate them into subgroups with the same morphological properties, allowing the identification of impurities and/or misrecognized structures (25). Another study evaluated functionalized gold nanoparticles as contrast agents for tumor imaging and employed ML algorithms to better evaluate and predict the biodistribution and toxicity of the nanoparticles in mice using the histological section images of tumors and some organs (26). Gold nanoparticles can also be used as immunolabeling in electron microscopy analysis to help in the identification of proteins and to observe their localization and density in the cells. A deep learning-based free software (Gold Digger), recently developed by Jerez and co-workers, permits to speed up and decrease annotating errors due to manual analysis of images for a more accurate outcome (27). Also, they are routinely used to screen for diseases in asymptomatic patients and to monitor the therapeutic progress (87). Advancements in technological infrastructures have given rise to the extensive use of computing in medical imaging. Today, computer-aided diagnosis and interventions support the identification of pathophysiological processes (88). For example, a study using tissue microarray technology coupled with optimized image analysis algorithms has successfully quantified 89% of tissue samples into breast malignancy and benign breast tissues respectively (89). To address the limitations in data evaluation, ML can be applied to provide new opportunities in image analysis. However, such investigations still require the preparation of a large number of tissue samples and are sometimes not available in early drug development (90).

Another aspect is the data density obtained with imaging methods. Each image is composed of millions of pixels organized in defined clusters. In this context, ML and AI can be used to identify patterns that are impervious to the operator (91). Incorporation of ML with an automated feature extraction classification algorithm into white blood cells detection models yields 95% classification accuracy. This provides an efficient workflow in medical diagnostics (92). In nanomedicine, modeling tools and ANNs have been successfully used to support cell response studies using time-lapse microscopy (28). The temporal cellular responses to RNA-based liposomes were measured using automated microscopy systems. The ANN was successfully trained to trace single cells and predict transfection efficiency (28). Another study evaluated cell internalization of nanoparticles as a diagnostic tool for the identification of breast cancer (29). Most cancer deaths arise from the formation of metastases. An ML-based image analysis associated with tissue clearing and 3D microscopy enabled the uptake of nanoparticles into micrometastases to be compared to primary tumors. Moreover, ML allowed an accurate prediction of the influence of the pathophysiology of micrometastases on nanoparticle delivery. This contributes to the identification of patient responses to specific treatments (30). The convolutional neural network U-Net was used to analyze videos obtained by liquid-phase transmission electron microscopy (93). Investigations of biodistribution processes using *in vivo* imaging techniques are often described in the literature and can be used for model validation and *in vitro-in vivo* correlation (94). In many cases, they often rely on conventional modeling approaches. However, more recent work reported the integration of near-infrared images for the investigation of nanoparticle penetration using a deep learning approach (32).

## Advantages and Limitations of Computational Aids

Today, computational aids are integrated into drug development at multiple levels. Their application in a modern production environment is widely based on the principles of QbD. Data collection using analytical sensors enables the process to be precisely controlled with a strong impact on the quality of the drug product. Also, excipient selection is often supported by complex statistical models and the data quantity even allows the use of ML algorithms and ANNs (6). With regard to nanomedicines, data quality plays a dominant role. At the nanoscale, some characteristics and differences between drug formulations are more difficult to measure. However, with the integration of more sophisticated analytical techniques for the determination of particle size or encapsulation efficiency as part of the IPCs, the control strategy becomes more effective. ML algorithms and ANNs provide an unbiased evaluation of process variables and can be used to determine their impact on the quality of the product as well as to predict functional and structural properties (e.g., drug release, cellular uptake, drug loading) and the degree of toxicity (95). In addition, computational aids can give support for the automatic production of nanoformulations, providing a quick process and increased yield (96). Still many studies emphasize well-understood processes and come with very similar conclusions compared to a conventional modeling approach. Another area of investigation is the *in vitro* and *in vivo* evaluation of nanomedicines including PK and PD studies. While PK studies often come with limited availability of data and smaller data sets, PD studies can rely on a number of *in vitro* screening methodologies. As a consequence, smart modeling approaches and simulations based on human-made computational frameworks are applied in PK analysis more successfully. By using a supervised neural network and *in vivo* protein corona formation data, Lazarovits et al. could predict with an accuracy up to 94% the half-life, liver and spleen accumulation, and size of PEGylated gold nanoparticles injected in rats. The biological fate of polymeric nanoparticles was also predicted, demonstrating the generalizable characteristic of the model (22). On the contrary, high-throughput screening and the automated analysis of biomolecules enabled the integration of effect-based screening and improved stratification into the characterization of PD. In this context, ML algorithms as well as ANNs sometimes support the identification of relevant effects. Among other examples, synergistic drug effects often depend on the exact concentration ratio at the target site and could be co-delivered by nanomedicines. A recent study demonstrated the advantage of using AI for identifying suitable drug combinations. The four anticancer drugs paclitaxel, nanodiamond-modified derivatives of doxorubicin, bleomycin, and mitoxantrone were used. The optimized drug-dose ratio simultaneously considered efficacy and safety of the combination treatment (23). With such synergisms depending on time, dose and patient, AI will have a pivotal role in optimizing drug combination therapies (97). In the future, they may even provide the only successful strategy in dealing with nanomedicine-related *omics'* data or information obtained from electronic sensors. AI will select the best targets as well as more personalized and precise treatments. Importantly, rapid computer-based analysis of large quantities of data is a unique feature of ML strategies. Consequently, recent developments in the early selection of drug delivery systems by high-throughput screening and the identification of efficacies depending on the interindividual differences between patient populations (as obtained from genomic, proteomic, or lipidomic analysis) would not have been possible without these new computational aids.

Although gifted with a considerable knowledge base, image analysis mainly focuses on cell interactions of nanomedicines. Only a few studies include more biodistribution data as well. One reason could be the complexity of *in vivo* images. Unbiased pattern recognition is likely to result in “hard-to-interpret” outcomes and is therefore limited to single cells and processes with a well-understood mechanism.

## Conclusion

In recent years, a rising number of computational aids has been applied in drug development. While the technical methods and algorithms are in place, their application in nanomedicine is widely driven by the availability of data. For drug products with a larger market share, preclinical and clinical studies are well-organized and study designs are widely comparable. Nanomedicines are characterized by a high complexity with regards to *in vitro* and *in vivo* performances (3). Therefore, the evolution of human-made computational models often leads to better understanding and, hence, significant progress in their development.

Whenever data is available in considerable quantity, e.g., due to the use of robotic *in vitro* platforms, automated analytical methods, or sensors, ML algorithms, and ANNs enable an unbiased evaluation. One critical component in the application of computational methods is the mechanistic understanding gained during data evaluation. In this context, the outcomes provided by ML sometimes do not comply with the established theoretical frameworks. As a consequence, whenever data sets come with high complexity, the outcomes may be very hard to interpret. Applications of AI in *in vivo* imaging are only one example where even small studies come with a high data volume, but the actual content of meaningful information is relatively low.

In conclusion, ML as well as modeling and simulation, both have their respective applications in nanomedicine development. “Big data” often leads to impressive insights identified by supervised ML algorithms. Whenever data sources are not accessible, computational frameworks and models may provide better answers. However, with more data becoming available a variety of evolving computational methods will support planning, design, process control, monitoring, and predictions, that lead to significant cost reduction and streamlined development processes. From a longer perspective, this will improve clinical translation and the availability of smart therapeutics such as nanomedicines.

## Author Contributions

MVN and TL drafted and revised the structure and most sections of the manuscript. SS, KJ, and SN contributed to selected subsections of the manuscript. RW and KC revised the draft manuscript. MW provided the concept, drafted selected sections, and revised the draft paper. All authors contributed to the article and approved the submitted version.

## Funding

MW was funded by the National University of Singapore and the Ministry of Education (grant no. R-148-000-282-133 and R-148-000-297-114).

## Conflict of Interest

RW was employed by YellowMap AG and KC was employed by isee systems. Both companies did not influence the content presented in this review article. The remaining authors declare that the research was conducted in the absence of any commercial or financial relationships that could be construed as a potential conflict of interest.

## Publisher's Note

All claims expressed in this article are solely those of the authors and do not necessarily represent those of their affiliated organizations, or those of the publisher, the editors and the reviewers. Any product that may be evaluated in this article, or claim that may be made by its manufacturer, is not guaranteed or endorsed by the publisher.
